# Maladaptive Plasticity for Motor Recovery after Stroke: Mechanisms and Approaches

**DOI:** 10.1155/2012/359728

**Published:** 2012-06-26

**Authors:** Naoyuki Takeuchi, Shin-Ichi Izumi

**Affiliations:** Department of Physical Medicine and Rehabilitation, Tohoku University Graduate School of Medicine, 2-1 Seiryo-cho, Aoba-ku, Sendai 980-8575, Japan

## Abstract

Many studies in human and animal models have shown that neural plasticity compensates for the loss of motor function after stroke. However, neural plasticity concerning compensatory movement, activated ipsilateral motor projections and competitive interaction after stroke contributes to maladaptive plasticity, which negatively affects motor recovery. Compensatory movement on the less-affected side helps to perform self-sustaining activity but also creates an inappropriate movement pattern and ultimately limits the normal motor pattern. The activated ipsilateral motor projections after stroke are unable to sufficiently support the disruption of the corticospinal motor projections and induce the abnormal movement linked to poor motor ability. The competitive interaction between both hemispheres induces abnormal interhemispheric inhibition that weakens motor function in stroke patients. Moreover, widespread disinhibition increases the risk of competitive interaction between the hand and the proximal arm, which results in an incomplete motor recovery. To minimize this maladaptive plasticity, rehabilitation programs should be selected according to the motor impairment of stroke patients. Noninvasive brain stimulation might also be useful for correcting maladaptive plasticity after stroke. Here, we review the underlying mechanisms of maladaptive plasticity after stroke and propose rehabilitation approaches for appropriate cortical reorganization.

## 1. Introduction

For several decades, many studies in both human and animal models have demonstrated that neural plasticity can change the structure and/or the function of the central nervous system after stroke and rehabilitation [1–3]. Although some neural plasticity undoubtedly contributes to motor recovery after stroke, it remains unclear whether all neural plasticities contribute to genuine motor recovery [1, 2, 4]. In addition to findings that neural plasticity aids in the acquisition of new skills and compensates for the loss of function [3, 5], it has been reported that injury and excessive training drive neural plasticity in a maladaptive direction [6, 7]. This neural plasticity is called “maladaptive plasticity,” which contributes to the pathogenesis of phantom pain and dystonia [6, 7]. Moreover, several studies have reported maladaptive plasticity weakens motor function and limits motor recovery after stroke [8–13].

The limbs contralateral to the side of the lesion exhibit hemiparesis after a motor stroke, and recovery of motor function after stroke is usually incomplete [14]. However, a large number (approximately 40–60%) of stroke patients can regain the ability to perform self-sustaining activities of daily living after rehabilitation therapy [15, 16]. Patients with stroke often develop a compensatory hyperreliance on the nonparetic side, proximal paretic side, or trunk movement to perform daily tasks [17–20]. The development of compensatory behaviors is an advantageous strategy, which permits the performance of daily activities despite motor impairments [21–23]. However, the strong and efficient motor compensations may prevent the affected side from generating normal motor patterns of daily activities [17, 23]. Moreover, its long-term neural and behavioral consequences are not well understood and may ultimately limit the final functional outcome.

The extent of functional gains from neural plasticity on the motor recovery of normal patterns or the compensatory movements of new patterns and the effect of rehabilitation on these processes are unclear [23]. Part of this problem is derived from the confusion consensus on the role of neural plasticity in motor recovery and compensatory movement [11, 23]. Therefore, it is important that neural plasticity resulting from compensatory movement is not misinterpreted as motor recovery [24]. Moreover, it is necessary to understand how brain activity and behavioral changes induce maladaptive plasticity after a stroke. This paper focuses on 4 factors that influence maladaptive plasticity in motor-related areas after stroke: (1) compensatory movement, (2) ipsilateral motor projections, (3) competitive interaction, and (4) rehabilitation and noninvasive brain stimulation. The purpose of this paper was to provide a comprehensive overview of maladaptive plasticity after stroke to understand its mechanisms and suggest the approaches for appropriate cortical reorganization.

## 2. Compensatory Movement after Stroke

First, the difference between motor recovery and compensatory movement must be clearly established. “Motor recovery” is defined as the reappearance of elemental motor patterns present before a stroke. In contrast, “compensatory movement” is defined as the appearance of new motor patterns resulting from the adaptation of remaining motor elements or substitution, meaning that functions are taken over, replaced, or substituted by different end effectors or body segments [23].

It is common in stroke patients with severe impairment that the compensatory or substitutive movements of the less-affected body side are encouraged to maximize functional ability [21–23]. In addition to their nonparetic side, patients with stroke often use their trunks and the proximal limb of their paretic side for compensatory movement, as they are less affected than the distal limb [25, 26]. In the upper limb, compensatory movement can include the use of motor patterns that incorporate trunk displacement and rotation, scapular elevation, shoulder abduction, and internal rotation [21, 27]. The use of compensatory movement can assist arm and hand transport and aid in hand positioning/orientation for grasping [28–30]. Also in the lower limb, stroke patients often use larger arm and leg swing amplitudes on the nonparetic side to increase walking speed [31]. Although compensatory movements may help stroke patients perform tasks in the short term, the presence of compensation may be associated with long-term problems such as reduced range of joint motion and pain [32]. Moreover, the increased activities of proximal arm due to compensatory movement may contribute to the abnormal interjoint movement that is often observed after a stroke [20].

In addition to a reduced range of joint motion and pain due to compensatory movement of the paretic limb, excessive use of the nonparetic limb can also induce another problem. Stroke patients often use the nonparetic limb instead of the paretic limb to perform daily activities. Dominant use of the nonparetic limb induces the phenomenon of learned nonuse of the paretic limb, which limits the capacity for subsequent gains in motor function of the paretic limb [23, 33]. Moreover, learned nonuse of the paretic limb induces the reduction of joint motion and more weakness in the paretic limb. It has been reported that the stroke lesion appears to facilitate the acquisition of new skills with the nonparetic limb in animal models [34, 35]. This might enable stroke patients to quickly resume some daily activities by compensatory strategies involving the nonparetic limb, but the easily acquired motor skills with the nonparetic limb might accelerate a pattern of learned nonuse of the paretic limb.

In addition to enhancement of learned nonuse of the paretic limb, it has been reported that skill acquisition with the nonparetic limb may negatively impact the experience-dependent plasticity of the affected hemisphere. In rats, motor training with the nonparetic limb reduces the neuronal transcription factor, which shows experience-dependent behavioral change in the affected hemisphere after training with the paretic limb [12]. The reason for this constraint of neuronal plasticity in the affected hemisphere by the nonparetic limb is not yet clear, but it may reflect experience-dependent alterations in interhemispheric activity [13, 36]. Thus, intense use of the nonparetic limb might have harmful effects on motor recovery of the paretic limb, and this is linked to reduced neuronal activation in the movement of the paretic limb and representations in the remaining cortex of the affected hemisphere. These findings suggest that the affected hemisphere becomes vulnerable to poststroke experience with the nonparetic limb and that this nonparetic limb experience may drive neural plasticity in a direction that is maladaptive for functional outcome [12]. Compensatory movement patterns may improve performance of daily activities after stroke but may also induce maladaptive plasticity and limit motor recovery.

## 3. Ipsilateral Motor Projections after Stroke

The contribution of ipsilateral motor projections to motor function after stroke has been evaluated mainly by transcranial magnetic stimulation (TMS) studies [37, 38]. Many of these studies have indicated that ipsilateral motor projections are enhanced after stroke [39–41]. Although the reason for this remains unclear, it is believed that latent ipsilateral motor projections are activated by disruption of the contralateral corticospinal projections in stroke patients [40, 42]. However, most studies on ipsilateral motor projections have reported negative results for motor function, especially for the distal side [40, 41]. The weak relationship between ipsilateral motor projections and motor function may be explained by the fact that distal muscles are primarily innervated by contralateral corticospinal projections [43], whereas ipsilateral motor projections to the distal muscles are scarce [44]. In addition to the upper limb, the strong ipsilateral motor projections to the paretic lower limb are correlated with poor motor function of ankle movement in stroke patients [45]. Thus, the ipsilateral motor projections might not be sufficient to support the disruption of the contralesional corticospinal projections to the distal side.

Furthermore, these ipsilateral motor projections to the paretic side might be not only unhelpful but also maladaptive for motor recovery in stroke patients. Although the dominant anatomical arrangements of the ipsilateral motor projections to the proximal muscles may contribute to the relative preservation of proximal limb control [44], it has been reported that the increased expression of ipsilateral motor projections to the paretic proximal side may contribute to the generation of abnormal interjoint coupling movement after stroke [20]. Given the smaller contralateral corticospinal input to the proximal limb, the subsequent expression of ipsilateral motor projections may explain the loss of independent joint control and abnormal interjoint movement observed in the proximal limb after stroke [20]. Because impairment of interjoint movement weakens the reaching abilities in stroke patients [46–48], enhancement of the ipsilateral motor projections to the paretic side might contribute to generation of an abnormal motor pattern leading to poor motor ability after stroke.

Despite the correlation between the expression of ipsilateral motor projections and poor motor ability after stroke described previously, the upregulation of ipsilateral motor projections may play an important role in preserving some degree of motor function, especially in children [49–51]. Moreover, the activation of ipsilateral motor projections may be beneficial for trunk muscle movement in more severely affected patients [52, 53]. Thus, the contribution of ipsilateral motor projections varies according to the clinical state of the patient. In stroke patients with relatively mildly affected motor function, the enhancement of ipsilateral motor projections may not be helpful, especially for the distal side, and induce an abnormal motor pattern linked to poor motor ability.

## 4. Competitive Interaction after Stroke

Stroke alters the neuronal function of the motor cortex adjacent to or distant from the lesion through neuronal networks [37]. TMS and functional magnetic resonance imaging studies have been used to detect the changes in neural function after stroke [37, 38, 54–57]. These changes in neuronal function are helpful for the loss of motor function; however, some changes may deteriorate the balance between neural networks and result in an incomplete motor recovery [11]. Several TMS studies have shown that the unaffected hemisphere inhibits the affected hemisphere through abnormal interhemispheric inhibition and restricts motor function after stroke [8, 9]. This hypothesis was also supported by reports that inhibitory stimulation over the unaffected hemisphere improves motor function rather than weakening it [58, 59]. Therefore, this interhemispheric competitive interaction is highlighted as a mechanism of maladaptive plasticity and is a treatment target for stroke [8, 58–60]. The mechanism of this interhemispheric competitive interaction is estimated to be the result of unbalanced changes in both hemispheres after stroke. It has been reported that stroke patients with poor motor function show more activation of the unaffected hemisphere [37, 55, 56]. Moreover, hyperexcitability of the unaffected hemisphere has a negative correlation with motor function after stroke [56, 61]. Therefore, in addition to the damage of the affected hemisphere by the stroke lesion, these changes in the unaffected hemisphere may lead to further interhemispheric unbalance, which induces abnormal interhemispheric inhibition and restricts motor recovery in stroke patients with poor motor function [37]. Moreover, the behavior pattern changes, like the compensatory usage of the nonparetic side, may promote the unbalance between the hemispheres [33].

In addition to interhemispheric competitive interaction, intrahemispheric competitive interaction is thought to induce maladaptive plasticity after stroke. From the viewpoint of inhibitory function, some studies have shown how changes in neural function can affect motor patterns after stroke [10, 62, 63]. The reduced inhibitory function is believed to be one of the mechanisms that contribute to neural plasticity by unmasking latent networks [64]. Therefore, disinhibition in the affected hemisphere may promote maladaptive plasticity by abnormal motor patterns of the paretic side, which often occurs in stroke patients. In fact, by using TMS, it has been reported that the inhibitory function of the ipsilesional premotor cortex (PMC) was disturbed in stroke patients whose hand function was poorer than their proximal arm function [10]. The motor projections from the PMC to the spinal cord are known to be less numerous and less excitatory than those from the M1 [65, 66]. Moreover, the projections from the PMC are more related to the control of muscle movements of the proximal arm [67, 68]. It has been reported that hand and the proximal arm regions compete for areas within the motor cortex [69]. Considering these findings, excitability, which is disproportionately distributed in the proximal arm because of weak inhibitory function of the PMC, might induce a competitive interaction between the hand and the proximal arm, resulting in the development of maladaptive plasticity in the hand.

**Figure 1:**
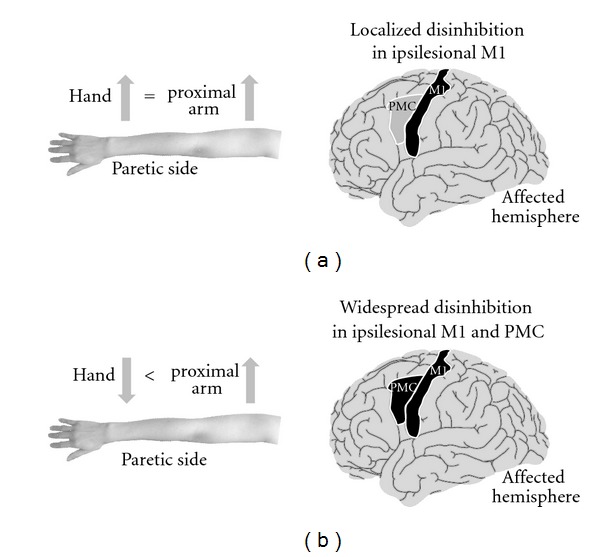
Maladaptive plasticity induced by disinhibition of motor-related areas in stroke patients. (a) Localized disinhibition in the ipsilesional primary motor cortex (M1). Localized disinhibition in the ipsilesional M1 promotes motor recovery by facilitating neural plasticity without competitive interaction between hand and proximal arm. (b) Widespread disinhibition in the ipsilesional M1 and premotor cortex (PMC). The disinhibition in the ipsilesional PMC causes uneven excitability distribution in the proximal arm and proximal-dominant competitive interaction in the ipsilesional M1 and PMC. As a result, this widespread disinhibition induces maladaptive plasticity that poorly controls the paretic hand in stroke patients.

Besides the PMC, the reduced inhibitory function of the ipsilesional M1 might induce competitive interaction between the hand and the proximal arm, because stroke patients often use the proximal arm for compensatory movement [23, 27]. However, it is unlikely that the unfavorable competitive interaction of the hand occurs in the ipsilesional M1 as well as in the PMC, because in the M1, the hand region is larger than proximal arm region [68, 70]. Moreover, a TMS study by using paired-pulse stimulation reported that the inhibitory function of the ipsilesional M1 is negatively correlated with the motor function of the paretic hand in stroke patients [62]. A recent study evaluating short-latency afferent inhibition also reported that the reduced inhibitory function of the ipsilesional M1 in acute stroke patients could promote motor recovery [63]. Therefore, the localized disinhibition of the ipsilesional M1 in stroke patients may promote the motor recovery of normal patterns by facilitating ipsilesional M1 plasticity. However, the widespread disinhibition of the affected hemisphere increases the risk of competitive interaction between the hand and the proximal arm, resulting in an incomplete motor recovery (Figure 1).

## 5. Approaches to Prevent Maladaptive Plasticity after Stroke

As described in the previous sections, compensatory movement may introduce maladaptive plasticity and limit genuine motor recovery after stroke. In particular, compensatory use of the nonparetic limb may inhibit learning new motor skills with the paretic limb. The excessive excitability of the unaffected hemisphere, activated by the use of the nonparetic limb, inhibits the affected hemisphere through abnormal interhemispheric inhibition. Moreover, the widespread disinhibition of the affected hemisphere might induce the competitive interaction that results in incomplete motor recovery. In this section, we propose approaches to prevent maladaptive plasticity after stroke.

### 5.1. Rehabilitation Programs

To prevent maladaptive plasticity after stroke, we should consider the competitive interaction hypothesis. In addition to competitive interaction between the proximal and distal sides, the increased activities of proximal limb due to compensatory movement itself may be associated with poor motor function and contribute to the abnormal interjoint movement that is observed following stroke [20]. Therefore, the rehabilitation program used may have to avoid intense training of the proximal side more than the distal side. However, to our knowledge, no rehabilitation program currently deals with this problem. Moreover, it is true that compensatory movement of the proximal muscle is useful for reaching in some stroke patients with poor motor function [23, 27]. Thus, at least in cases where stroke patients have good motor function, a rehabilitation program may be helpful in avoiding compensatory use of the proximal side according to the competitive interaction hypothesis. This problem can be eliminated, at least in part, if regional anesthesia of the upper arm during hand motor practice could potentiate practice-induced improvements in hand motor function in chronic stroke patients [69]. Further investigation is required to clarify the effect of a rehabilitation program on the competitive interaction between the proximal and distal sides.

Considering the competitive interaction between the paretic and nonparetic sides, it is natural that the rehabilitation program should avoid nonuse of the paretic limb. In human stroke survivors, the disability of the paretic arm leads to its disuse, which limits functional improvement, a phenomenon termed “learned nonuse” [33]. The facilitation of neural plasticity underlying compensatory learning with the nonparetic limb after stroke can also exacerbate the learned nonuse via abnormal interhemispheric inhibition [13, 36]. However, it is possible that longer training of the paretic limb could overcome the maladaptive effects of prior nonparetic limb experience and learned disuse of the paretic limb [36]. Particularly, the constraint-induced movement therapy (CIMT) that combines a rehabilitative training regime for the paretic limb with constraint of the nonparetic limb can overcome learned nonuse of the paretic limb and has been shown to improve motor function in animal models and stroke patients [33, 71–73]. Moreover, it has been reported that CIMT improves the imbalance in both hemispheres after stroke [74]. Therefore, clinicians should consider the CIMT for stroke patients who fit its criteria to facilitate appropriate reorganization.

Studies on animal stroke models suggest that compensatory use of the nonparetic limb while the paretic limb is being used does not necessarily induce the maladaptive change of learned nonuse [12]. Therefore, bilateral training therapy may be effective in preventing the learned nonuse of the paretic side. In humans, bilateral movement training improves the balance of excitability in both hemispheres [75, 76] and is effective for improving motor function in stroke patients [77]. However, bilateral training may facilitate more recruitment of ipsilateral motor projections [78, 79] and thus may be more advantageous for proximal arm function than for hand function [80]. Considering these reports, bilateral training may prevent learned nonuse but enhance maladaptive plasticity of the distal side. Therefore, different rehabilitation programs should be selected according to motor impairment. Stroke patients with good motor function might do better in performing intense training of the paretic limb such as the CIMT. In contrast, stroke patients with poor motor function who are compelled to the compensatory use of the nonparetic limb in daily activity have the advantage in bilateral movement training to prevent learned nonuse [81], although maladaptive plasticity of the distal side might occur. Future studies are needed to clarify the possible effects of bilateral movement training for maladaptive plasticity after stroke.

### 5.2. Noninvasive Brain Stimulation

Repetitive TMS (rTMS) and transcranial direct current stimulation (tDCS) are noninvasive brain stimulation (NIBS) techniques that can alter the excitability of the human cortex for several minutes [82]. Many reports have shown that NIBS improves neurological disorders by using their physiological peculiarity [82, 83]. In addition to the disruption of corticospinal motor projections from the affected hemisphere, the affected hemisphere is disturbed by the unaffected hemisphere via interhemispheric inhibition in stroke patients [8, 9, 58]. This interhemispheric competition model proposes that motor deficits in stroke patients are due to reduced output from the affected hemisphere and excessive interhemispheric inhibition from the unaffected hemisphere to the affected hemisphere. Considering the interhemispheric competition model, improvement in motor deficits could be achieved by increasing the excitability of the affected hemisphere or decreasing the excitability of the unaffected hemisphere using NIBS [60, 84].

It has been reported that experience-dependent plasticity is impaired in the affected hemisphere [85, 86]; however, NIBS may solve this problem by facilitating plasticity in the affected hemisphere. Pairing of rehabilitative training with NIBS results in more enduring performance improvements and functional plasticity in the affected hemisphere compared with motor training or stimulation alone [58–60, 84]. Moreover, it has been reported that NIBS could induce long-term potentiation-like changes in the affected hemisphere after stroke and promote motor recovery [61]. In addition to the facilitation of experience-dependent plasticity, NIBS may prevent the negative effect of nonparetic limb training after stroke. Learning a skilled motor task with the nonparetic limb worsens performance and relearning with the paretic limb [12, 87]. This maladaptive effect was absent in animals with transections of the corpus callosum [36]. It has also been reported that inhibitory NIBS reduced interhemispheric interaction in stroke patients [58, 88]. Moreover, excitatory NIBS over the affected hemisphere produces long-term depression-like changes in the unaffected hemisphere [63]. Therefore, NIBS may prevent the maladaptive plasticity produced by nonparetic movement, which worsens motor learning with the paretic limb. Future investigation is required to clarify whether NIBS ameliorates maladaptive plasticity caused by compensatory movement of the nonparetic limb.

**Figure 2:**
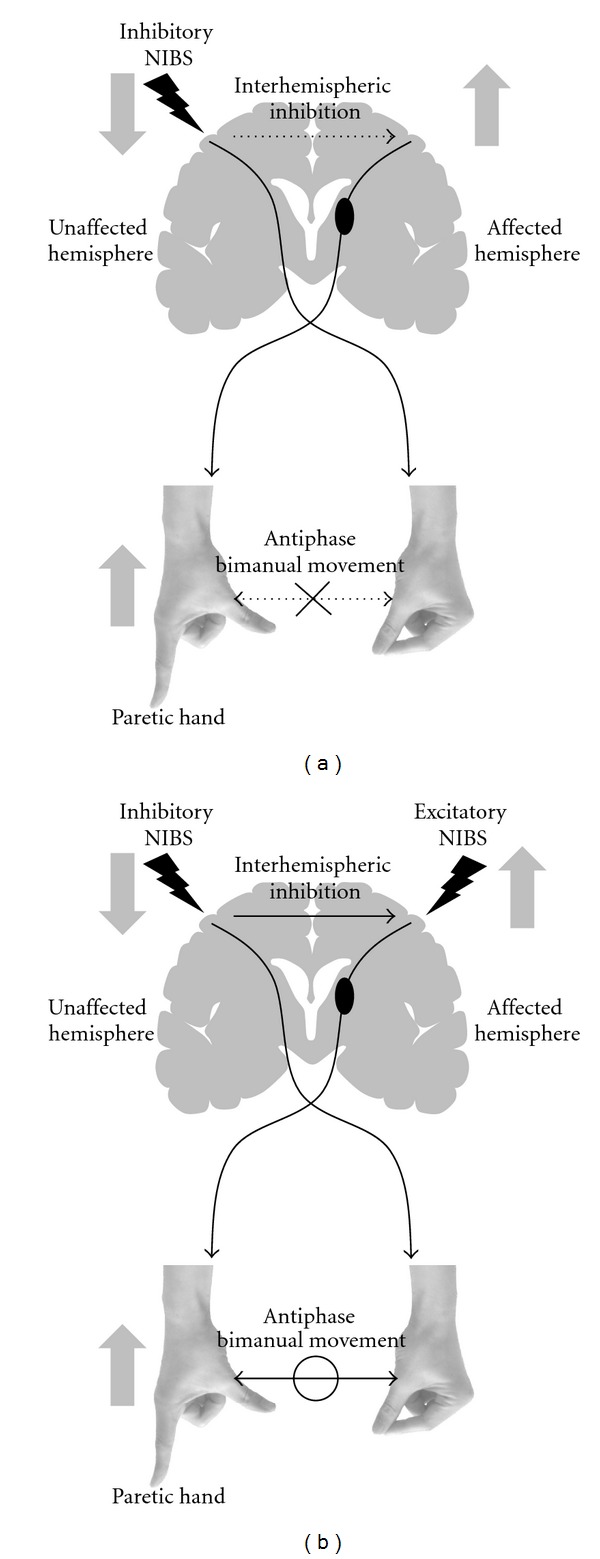
Mechanism of motor function change after noninvasive brain stimulation (NIBS) in stroke patients. (a) Inhibitory NIBS over the unaffected hemisphere. Inhibitory NIBS decreases excitability of the contralesional motor cortex (M1) and increases excitability of the ipsilesional M1 by reducing interhemispheric inhibition from the unaffected to the affected hemisphere. Facilitation of the ipsilesional M1 improves motor function of the paretic hand in stroke patients. However, the antiphase bimanual movement deteriorates owing to the reduction of interhemispheric inhibition, which controls bimanual movement. (b) Bilateral NIBS. Excitatory NIBS along with inhibitory NIBS also decreases excitability of the contralesional M1, increases excitability of the ipsilesional M1, and improves motor function of the paretic hand in stroke patients. Bilateral NIBS lessens the reduction of interhemispheric inhibition induced by inhibitory NIBS and prevents deterioration of antiphase bimanual movement. Modified from Takeuchi et al. [88].

Although NIBS may be useful to prevent maladaptive plasticity by correcting abnormal interhemispheric inhibition and facilitation of experience-dependent plasticity in the affected hemisphere, it must be noted that the NIBS itself also induces maladaptive plasticity after stroke. A recent study has reported that the inhibitory rTMS over the unaffected hemisphere led to deterioration of the antiphase bimanual movement in stroke patients [88]. Inhibitory NIBS might worsen the antiphase bimanual movement by reducing the interhemispheric inhibition that controls bimanual movement [89, 90] (Figure 2(a)). However, a combination of inhibitory NIBS over the unaffected hemisphere and excitatory NIBS over the affected hemisphere could prevent the deterioration of bimanual movement by lessening the reduction of interhemispheric inhibition (Figure 2(b)) [88]. It has been suggested that inhibitory interneurons in the affected hemisphere activated by excitatory NIBS may lessen the reduction of interhemispheric inhibition from the unaffected to the affected hemisphere in a bilateral NIBS protocol [88]. Moreover, it has been reported that bilateral NIBS using rTMS improves motor function more effectively than unilateral rTMS by inducing the excitability and the disinhibition of the ipsilesional M1 [84]. In addition to rTMS studies, bilateral tDCS methods have been reported to be more effective in improving motor function after stroke than unilateral tDCS [91, 92]. Therefore, clinicians can also consider bilateral NIBS as part of the rehabilitation program to improve motor function and prevent maladaptive plasticity in stroke patients.

## 6. Conclusion

This paper focuses on the underlying mechanisms of maladaptive plasticity, which inhibits motor recovery after stroke, and the approaches that can be used to solve this problem. Compensatory movements may help stroke patients perform tasks in the short term but may also be associated with long-term problems such as learned nonuse, reduced range of joint motion, and pain. Moreover, compensatory movement of the nonparetic limb may induce maladaptive plasticity of the affected hemisphere and limit motor recovery after stroke. Activation of ipsilateral motor projections may be beneficial for trunk muscle movement, more severely affected patients and children. However, enhancement of ipsilateral motor projections may also be detrimental for the distal side and may induce abnormal movement patterns linked to poor motor ability. Stroke causes unbalanced excitability between both hemispheres and results in abnormal interhemispheric inhibition from the unaffected hemisphere to the affected hemisphere; this restricts motor function in stroke patients. Moreover, neural plasticity caused by localized disinhibition in the ipsilesional M1 is appropriate for motor recovery; however, widespread disinhibition increases the risk of competitive interaction between the hand and the proximal arm, which results in an incomplete recovery. To prevent this maladaptive plasticity, it is necessary to avoid learned nonuse and excessive use of compensatory movement. The NIBS technique ameliorates maladaptive plasticity by facilitating experience-dependent plasticity and correcting abnormal interhemispheric inhibition. However, it must be noted that inhibitory NIBS over the unaffected hemisphere itself might induce another maladaptive plasticity that deteriorates bimanual movement. The new method of bilateral NIBS can prevent deterioration of bimanual movement and facilitate motor function more than unilateral NIBS can because of its ability to induce disinhibition in the ipsilesional M1 and correct the imbalance between the hemispheres. Future studies should focus on better understanding the effects of rehabilitation and NIBS on maladaptive plasticity after stroke.
